# Supplementary material for: Causal Relationship between Aging and Anorexia Nervosa: A White-Matter-Microstructure-Mediated Mendelian Randomization Analysis
Source: Biomedicines. 2024 Aug 16;12(8):1874. doi: 10.3390/biomedicines12081874 (PMC11351342; doi:10.3390/biomedicines12081874)
Supplement: Supplementary file 1 [file biomedicines-12-01874-s001.zip › Supplementary Figure/Figure S9/Leaveoneout plot of MR analysis for ubm-b-1946 to AN.pdf]

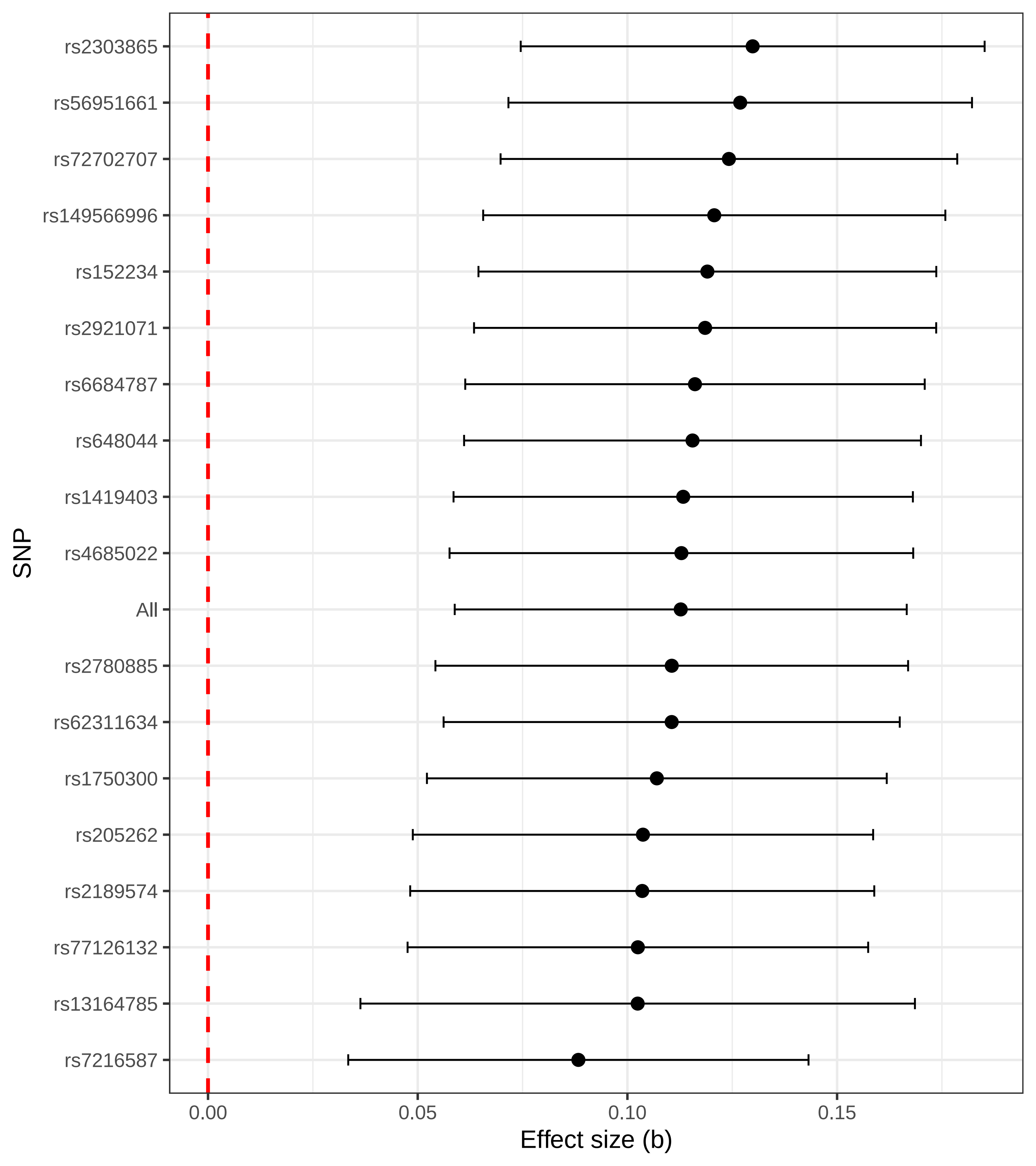
rs2303865
rs56951661
rs72702707
rs149566996
rs152234
rs2921071
rs6684787
rs648044
rs1419403
rs4685022
All
rs2780885
rs62311634
rs1750300
rs205262
rs2189574
rs77126132
rs13164785
rs7216587
SNP
0.00
0.05
0.10
0.15
Effect size (b)
